# The potential dual role of tau phosphorylation: plasma phosphorylated-tau217 in newborns and Alzheimer’s disease

**DOI:** 10.1093/braincomms/fcaf221

**Published:** 2025-06-07

**Authors:** Fernando Gonzalez-Ortiz, Jakub Vávra, Emma Payne, Bjørn-Eivind Kirsebom, Ulrika Sjöbom, Cristiano Santos, Jordi Júlvez, Kaitlin Kramer, David Zalcberg, Laia Montoliu-Gaya, Michael Turton, Peter Harrison, Ann Hellström, Henrik Zetterberg, Tormod Fladby, Marc Suárez-Calvet, Robert D Sanders, Kaj Blennow

**Affiliations:** Department of Psychiatry and Neurochemistry, Institute of Neuroscience and Physiology, The Sahlgrenska Academy at the University of Gothenburg, Gothenburg 417 11, Sweden; Clinical Neurochemistry Laboratory, Sahlgrenska University Hospital, Mölndal 431 80, Sweden; Neurology Department, Neurocode USA Inc, Bellingham, WA 98225, USA; Department of Psychiatry and Neurochemistry, Institute of Neuroscience and Physiology, The Sahlgrenska Academy at the University of Gothenburg, Gothenburg 417 11, Sweden; St George Hospital, South Eastern Sydney Local Health District, Sydney, New South Wales 2217, Australia; Department of Neurology, University Hospital of North Norway, Tromsø 9038, Norway; Department of Psychology, Faculty of Health Sciences, The Arctic University of Norway, Tromsø 9037, Norway; Department of Neurology, Akershus University Hospital, Lørenskog 1478, Norway; Learning and Leadership for Health Care Professionals at the Institute of Health and Care Science at Sahlgrenska Academy at University of Gothenburg, Gothenburg 417 11, Sweden; Department of Clinical Neuroscience at the Institution of Neuroscience and Physiology at Sahlgrenska Academy at University of Gothenburg, Gothenburg 417 11, Sweden; Clinical Neurochemistry Laboratory, Sahlgrenska University Hospital, Mölndal 431 80, Sweden; Clinical and Epidemiological Neuroscience Group (NeuroÈpia), Institut d’Investigació Sanitària Pere Virgili (IISPV), Reus 43204, Spain; Central Clinical School, Faculty of Medicine and Health, The University of Sydney, Sydney, New South Wales 2006, Australia; Department of Anaesthetics, Royal Prince Alfred Hospital, Sydney Local Health District, Sydney, New South Wales 2050, Australia; Central Clinical School, Faculty of Medicine and Health, The University of Sydney, Sydney, New South Wales 2006, Australia; Department of Anaesthetics, Royal Prince Alfred Hospital, Sydney Local Health District, Sydney, New South Wales 2050, Australia; Department of Psychiatry and Neurochemistry, Institute of Neuroscience and Physiology, The Sahlgrenska Academy at the University of Gothenburg, Gothenburg 417 11, Sweden; Bioventix Plc, Farnham, Surrey GU9 7SX, UK; Bioventix Plc, Farnham, Surrey GU9 7SX, UK; Department of Clinical Neuroscience at the Institution of Neuroscience and Physiology at Sahlgrenska Academy at University of Gothenburg, Gothenburg 417 11, Sweden; Department of Psychiatry and Neurochemistry, Institute of Neuroscience and Physiology, The Sahlgrenska Academy at the University of Gothenburg, Gothenburg 417 11, Sweden; Clinical Neurochemistry Laboratory, Sahlgrenska University Hospital, Mölndal 431 80, Sweden; Department of Neurodegenerative Disease, UCL Institute of Neurology, London WC1N 3BG, UK; UK Dementia Research Institute at UCL, London WC1N 3BG, UK; Hong Kong Center for Neurodegenerative Diseases, Hong Kong 200020, China; Wisconsin Alzheimer’s Disease Research Center, University of Wisconsin School of Medicine and Public Health, University of Wisconsin-Madison, Madison, WI 53 792, USA; Department of Neurology, Akershus University Hospital, Lørenskog 1478, Norway; University of Oslo, Institute for Clinical Medicine, Campus Ahus, Oslo 1478, Norway; Barcelonaβeta Brain Research Center (BBRC), Pasqual Maragall Foundation, Barcelona 08005, Spain; Hospital del Mar Research Institute, Barcelona 08003, Spain; Servei de Neurologia, Hospital del Mar, Barcelona 08003, Spain; Central Clinical School, Faculty of Medicine and Health, The University of Sydney, Sydney, New South Wales 2006, Australia; Department of Anaesthetics, Royal Prince Alfred Hospital, Sydney Local Health District, Sydney, New South Wales 2050, Australia; NHMRC Clinical Trials Centre, The University of Sydney, Sydney, New South Wales 2006, Australia; Institute of Academic Surgery, Royal Prince Alfred Hospital, Sydney Local Health District, Sydney, New South Wales 2050, Australia; Department of Psychiatry and Neurochemistry, Institute of Neuroscience and Physiology, The Sahlgrenska Academy at the University of Gothenburg, Gothenburg 417 11, Sweden; Clinical Neurochemistry Laboratory, Sahlgrenska University Hospital, Mölndal 431 80, Sweden; Paris Brain Institute, ICM, Pitié-Salpêtrière Hospital, Sorbonne University, Paris 75013, France; Neurodegenerative Disorder Research Center, Division of Life Sciences and Medicine, and Department of Neurology, Institute on Aging and Brain Disorders, University of Science and Technology of China and First Affiliated Hospital of USTC, Hefei 230026, China

**Keywords:** phosphorylated tau, newborns, plasma biomarkers, Alzheimer’s disease

## Abstract

Tau phosphorylation plays an important role in brain physiology and pathology. During foetal development, it supports microtubule dynamics and neuroplasticity, whereas in Alzheimer’s disease (AD), it drives pathological tau aggregation and tangle formation. In this multicentre study (*n* = 462), we measured plasma phosphorylated-tau217 in healthy newborns, premature infants, patients with AD and healthy controls across various age groups. Plasma phosphorylated-tau217 levels were significantly higher in newborns compared to healthy individuals of any age group and even exceeded levels observed in patients with AD. In newborns, plasma phosphorylated-tau217 levels inversely correlated with perinatal factors such as gestational age. Longitudinal analysis of preterm infants demonstrated a decline in serum phosphorylated-tau217 levels over the first months of life, approaching levels observed in young adults. In contrast, elevated plasma phosphorylated-tau217 in older individuals was associated with AD pathology. Our findings corroborate the crucial role of tau phosphorylation in early brain development. However, in AD, tau phosphorylation transitions into a pathological mechanism. The high levels of blood-based phosphorylated-tau217 observed at birth and subsequent clearance might indicate distinct regulatory mechanisms that prevent tau aggregation in early life. Further studies are needed to explore the shared mechanisms of tau phosphorylation in newborns and AD.

## Teaser

Gonzalez-Ortiz *et al*. report measurements of plasma phosphorylated-tau217 across different age groups. Levels were significantly higher in newborns than in patients with Alzheimer’s disease or individuals from any other age group. In premature newborns, elevated serum phoshorylated-tau217 levels persisted for up to 18 weeks after birth.

## Graphical Abstract

**Graphical Abstract:**
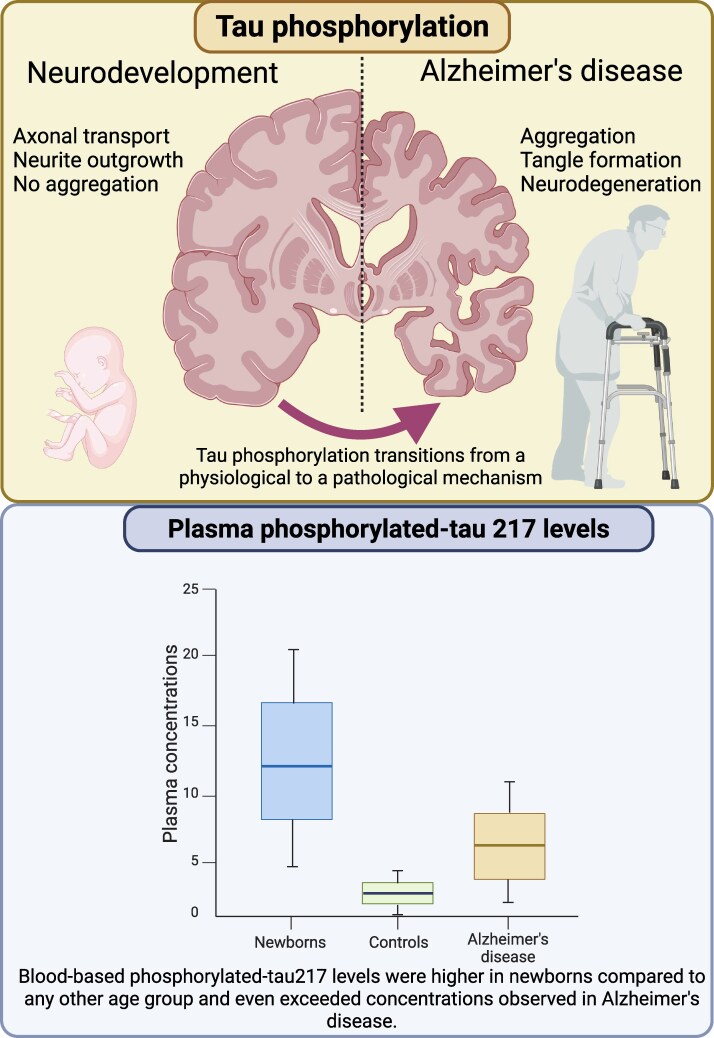

## Introduction

Tau is a critical protein involved in regulating microtubule dynamics, axonal transport and neurite outgrowth, with each function being modulated by phosphorylation at specific sites.^1,2^ In the foetal brain, tau is predominantly expressed as a shorter isoform lacking exon 10, which results in a form with three microtubule-binding repeats (3R tau).^3^ In contrast, the adult brain expresses a mixture of both 3R and 4R tau isoforms, the latter including exon 10, which adds a fourth microtubule-binding region.^3^ In addition to differences in microtubule-binding repeats, foetal tau predominantly contains zero N-terminal inserts (0N), while adult tau includes all six isoforms with varying numbers of N-terminal repeats (1N, or 2N), contributing to its functional diversity.^3,4^

Dysregulations in tau phosphorylation are strongly implicated in tau dysfunction,^1,5^ a key feature in Alzheimer’s disease, where abnormal phosphorylation contributes significantly to disease progression.^6^ In Alzheimer’s disease, tau phosphorylation not only leads to a loss of function but may also introduce harmful gain-of-function effects, such as making tau prone for aggregation with the subsequent development of tangle pathology.^7-9^ In contrast, in the developing foetal brain, high phosphorylated tau (p-tau) levels correlate with dynamic microtubule activity during key periods of brain plasticity and supports healthy neuronal development without forming tangles.^10,11^ Moreover, increased p-tau concentrations in CSF from newborns have been reported.^12^ However, it is unknown whether the heightened phosphorylation of tau observed in foetal brains and CSF from newborns can also be detected in peripheral blood using the currently available immunoassays and whether plasma p-tau levels can serve as clinically relevant markers in newborns.

Levels of plasma p-tau, particularly p-tau217, have been effectively used in blood-based assays to identify Alzheimer’s disease pathology even in the disease’s preclinical stages.^13,14^ Due to a clear increase in Alzheimer’s disease and strong association with amyloid markers, especially amyloid PET positivity, the term ‘amyloid-dependent’ is often applied to describe blood-based p-tau markers.^15,16^ Plasma p-tau217 also correlates with increasing severity of tau pathology measured by tau PET^17^ and tangle load at autopsy^18^ serving not only as a diagnostic but also as a prognostic marker in Alzheimer’s disease.^19^ However, the dependency of p-tau to amyloid has been debated extensively.^20-22^ For instance, we and other groups have shown that plasma p-tau can be an amyloid-independent marker in neurodegenerative disorders such as Niemann–Pick disease type C, Creutzfeldt–Jakob disease and amyotrophic lateral sclerosis, and that a temporary increase in plasma p-tau is seen also in acute neurological conditions such as traumatic brain injury and cardiac arrest, with higher levels predicting poorer clinical long-term outcomes.^22-27^ Altogether, these findings suggest that tau phosphorylation serves a more complex role beyond its association with Alzheimer’s disease pathology.^20^

Understanding the key physiological brain mechanisms that protect the foetal brain from phosphorylated tau-related pathology may offer insights into therapeutic strategies to prevent or reverse tau pathology in neurodegenerative diseases.^4,11^ Here, we aim to investigate phosphorylated tau in newborns and Alzheimer’s disease by comparing levels of blood-based p-tau217 in different age groups and Alzheimer’s disease.

## Materials and methods

Cohort-1 (ALFA age): The use of umbilical cord blood samples was approved by the Ethics Committee (CEIm) of Vall d’Hebron University Hospital on 27 July 2018, and by the scientific committee and the scientific management of the Banc de Sang i Teixits (BST) on 16 July 2018. This study was approved by the Independent Ethics Committee ‘Parc de Salut Mar,’ Barcelona (2015/6026/I). The ALFA Age study (2018/8089/I) was approved by the Independent Ethics Committee ‘Parc de Salut Mar,’ Barcelona.

Cohort-2: Ethics approval was obtained from SLHD Research and Ethics Committee [Royal Prince Alfred Hospital (RPAH) zone]. Trained recruiters obtained written, informed consent in English for all maternal participants in antenatal visits or on the birthing unit. Participants were excluded if they were non-English speaking, or had a history of psychological illness or other conditions that may interfere with capacity to provide informed consent after appropriate counselling in English. The study was performed according to the National Statement on Ethical Conduct in Human Research (2007) (link to national statement) and the CPMP/ICH Note for Guidance on Good Clinical Practice (link to CPMP/ICH); approval number: 2022/ETH01100.

Cohort-3 (DDI) has been approved by the Regional Committees for Medical and Health Research Ethics in Norway (REK: 2013/150). All participants gave a written informed consent before participating in the study.

Cohort-4 (premature newborns) from a randomized controlled trial was approved by the Regional Ethical Board in Gothenburg (Dnr 303-11) and registered at ClinicalTrials.gov (NCT02760472). Infants were included after oral and written informed consent from parents or legal guardians.

### Participant characteristics

#### Cohort-1: ALFA age

Cohort-1 is an observational and cross-sectional study conducted at the Barcelonaβeta Brain Research Center (BBRC), and its main aim was to assess the effect of aging in blood biomarkers in different age groups. This study compared four age groups: (i) umbilical cord blood from newborns; (ii) plasma from teenagers (12–17 years old); (iii) plasma from young adults (18–25 years old); and (iv) plasma from old adults (≥70). The human umbilical cord blood was obtained from the Catalan blood and tissue bank. The inclusion criteria of the study was the following: (i) males and females between 18 and 25 years (young adults group) or ≥70 years for persons at the time of inclusion (old adults group); (ii) subjects with no subjective cognitive complaints; (iii) individuals interested in participating in the study who fully understand all the procedures that will be performed; (iv) explicit participant agreement to undergo all the study procedures, which encompass: collection of basic demographic data and collection of a blood sample; and (v) give informed consent and agree that no data resulting from the study (which is no clinically relevant) will be given to the participant. The exclusion criteria were the following: (i) cognitive impairment shown by a MMSE < 26; (ii) any significant systemic illness or unstable medical condition that could lead to difficulty complying with the protocol [including auditive and visual impairment, renal insufficiency under haemodialysis treatment, hepatic cirrhosis, chronic pneumopathy under oxygen therapy, solid organ transplantation, fibromyalgia, active oncologic disease under treatment (excluding localized tumours)]; (iii) any significant major psychiatric illness (following DSM-IV diagnosis manual) or diseases that interfere with cognitive function (including major depression disorder, bipolar disorder, schizophrenia); (iv) acquired brain injury: brain traumatic injury with parenchymal or extra-axial macroscopic injury, large vessel ischaemic stroke or haemorrhagic stroke, brain tumours or other conditions that may cause acquired brain injury (brain radio- or chemotherapy); (v) Parkinson’s disease, epilepsy under treatment and with frequent seizures (>1/month) in the last year, multiple sclerosis or any other neurodegenerative disease; and (vi) researcher criteria: individuals that have any condition that, under researcher’s view, could lead to difficulty complying with the protocol. All participants in the ALFA Age study signed the study’s informed consent form that had also been approved by the Independent Ethics Committee ‘Parc de Salut Mar,’ Barcelona.^28^ Demographic characteristics of the participants can be found in Table 1.

**Table 1:** Demographic information for cohorts 1–3

ALFA age cohort			
	Teenagers	Young adults	Older adults
	(12)	(28)	(28)
Age, years	NA	20.9 (1.9)	73.9 (1.9)
Mean (SD) [range]		[19–26]	[70–77]
Female, *n* (%)	6 (50.0)	10 (35.7)	13 (46.4)
Plasma p-tau217 pg/mL, mean (SD)	1.8 (0.4)	1.4 (0.7)	1.8 (1.3)

DDI cohort			
	Controls A− (56)	MCI A+ (147)	Dementia A+ (16)
Age, years	61.2 (9.8)	67.7 (8.0)	66.6 (8.9)
Mean (SD) [range]	[41–80]	[50–83]	[51–83]
Female, *n* (%)	35 (62.5)	76 (51.7)	6 (37.5)
MMSE, median (IQR)	30 (1)	27 (3)	22 (4)
Plasma p-tau217 pg/mL, mean (SD)	1.7 (0.7)	3.6 (1.65)	4.6 (2.4)

Newborns		
	ALFA age cohort	RPAH cohort
	(55)	(106)
Gestational age (weeks), mean (SD)	39.7 (0.8)	38.2 (2.2)
Female, *n* (%)	30 (54.5)	NA
Plasma p-tau217 pg/mL, mean (SD)	10.2 (3.9)	9.2 (4.9)

A+/−, positive or negative CSF marker for amyloid plaques; N+/−, positive or negative marker for neurodegeneration; MCI, mild cognitive impairment; SD, standard deviation; IQR, interquartile range; *n*, number of cases; %, percentage; DDI, Dementia Disease Initiation; RPAH, Royal Prince Alfred Hospital.

#### Cohort-2: RPAH

In cohort-2, umbilical venous cord blood was collected by a trained attending midwife in the immediate postpartum period, either on the birthing unit or in operating theatres. A total of 1–3 mL samples were collected in addition to standard samples and centrifuged in the RPAH Department of Anaesthetics laboratory, and plasma samples were then stored in deidentified cryovials. The inclusion and exclusion criteria are described in an earlier study.^29^ Demographic characteristics of the participants can be found in Table 1.

#### Cohort-3: dementia disease initiation

Healthy controls and Alzheimer’s disease patients for the current project were sourced from the Norwegian Dementia Disease Initiation (DDI) cohort database. The Norwegian multicentre study DDI cohort comprise non-demented participants aged between 40 and 80 years with a native language of either Norwegian, Danish or Swedish. Participants were primarily recruited from memory clinics and advertisements in local news media, recruited at university hospitals across Norway between 2013 and 2022. Demographic characteristics of the participants can be found in Table 1. A detailed description of inclusion and exclusion criteria is outlined in a previous publication.^30^

#### Cohort-4: premature newborns

Cohort-4 includes 14 out of 90 extremely preterm infants (<28 weeks gestational age) from a randomized interventional study at the Queen Silvia Children’s Hospital neonatal intensive care unit between April 2013 and September 2015. Infants were randomized to different parenteral nutrition strategies regarding lipids, either an omega-3-enriched lipid emulsion (SMOF-lipid) or standard care (Clinoleic). Blood samples were collected as serum from the umbilical cord and at postnatal Days 1, 7, 14 and 28 and at postmenstrual ages 32, 36 and 40 weeks. The inclusion and exclusion in the study are described earlier.^31^

### Sample collection and biomarker measurements

Plasma and serum samples were obtained according to standard procedures and stored at −80°C until use. Blood-based p-tau217 on the Simoa HD-X platform using the University of Gothenburg in-house protocol was previously described.^13^ In cohort-1, plasma neurofilament light chain (NfL), total tau, amyloid42 and amyloid40 were measured using Quanterix commercial kits (103670) in a subset of participants. Plasma brain-derived tau (BD-tau) in cohort-2 was measured using the Gothenburg University in-house method.^32^ Plasma p-tau217 measurements in cohorts 1–4 were performed between January and September 2024. Signal variations within and between analytical runs were assessed using two internal quality control plasma samples at the beginning and the end of each run.

### Statistical analysis

Statistical analyses were performed with Prism, version 9.3.1 (GraphPad) and RStudio (R version 4.3.2). The distributions of data sets were examined for normality using the Kolmogorov–Smirnov test. Because the data were nonnormally distributed, nonparametric tests were used. *P*-values (including those adjusted for multiple comparisons) were considered significant at the two-sided *P* < 0.05 level. Fold changes were examined by comparing biomarker values with the means of the control groups.

## Results

### Cohort characteristics

‘Cohort-1’ was the ALFA age cohort (*n* = 123) that included individuals in different age groups (newborns, *n* = 55; teenagers, *n* = 12; young adults, *n* = 28; older adults, *n* = 28). Cross-sectional analyses in this cohort allowed us to investigate the group differences in levels of plasma p-tau217 at different ages. The plasma p-tau217 results in cohort-1 were independently validated in a clinical cohort (‘cohort-2’), which consisted of neonatal patients from the RPAH, Sydney (*n* = 106). Moreover, plasma p-tau217 results in cohort-1 and -2 were compared against the Dementia Disease Initiation cohort (‘cohort-3’) that included plasma samples from individuals with CSF positive biomarkers for Alzheimer’s disease with either mild cognitive impairment or Alzheimer’s disease dementia (*n* = 163) and controls (*n* = 56). Alzheimer’s disease was defined by CSF biomarker positivity (A^+^, as defined by CSF Aβ42/40 < 0.077). Finally, ‘cohort-4’ included serum samples from premature newborns (*n* = 14) with longitudinal measurements up to Day 133 after birth. The analyses performed in this cohort aimed to investigate longitudinal changes in serum p-tau217 after birth in premature newborns. Demographic characteristics for cohort-1–3 can be found in demographic Table 1. Demographic information for cohort-4 can be found in Supplementary Table 1. Additionally, Cohort 1 included an additional set of samples (*n* = 111) with biomarker data for plasma NfL, total tau, Aβ42 and Aβ40. However, due to volume constraints, plasma p-tau217 levels were not measured in this sample set, precluding direct comparisons between p-tau217 and other biomarkers. The demographic characteristics of these participants are presented in Supplementary Table 2.

### Plasma p-tau217 levels across age groups

**Figure 1:**
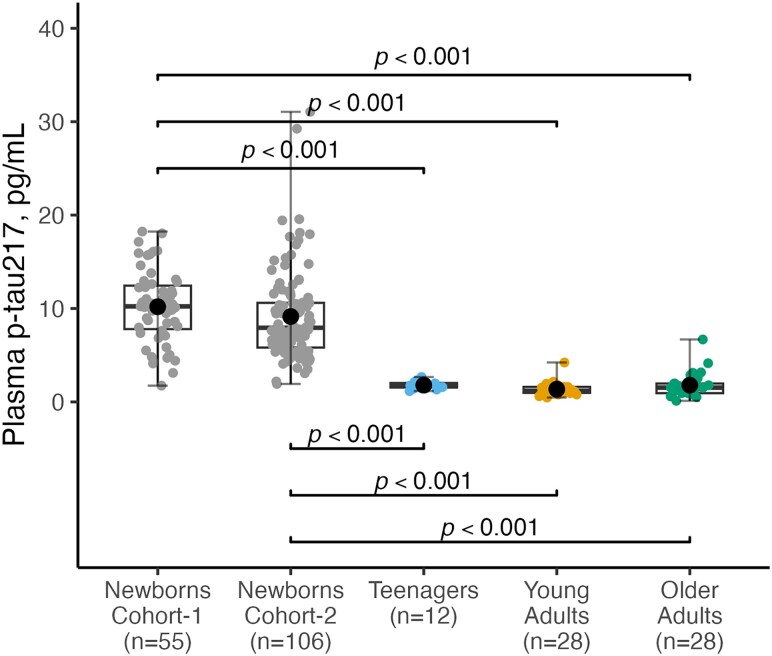
**Plasma p-tau217 levels across different age groups.** The figure shows a comparison of plasma p-tau217 concentrations (pg/mL) in newborns from cohort-1 (*n* = 55), newborns from cohort-2 (*n* = 106), teenagers (*n* = 12), young adults (*n* = 28) and older adults (*n* = 28). Group differences were examined using the Mann–Whitney test (two categories) or the Kruskal–Wallis test with Dunn’s multiple comparisons (three groups). In each box plot, the horizontal bar on top of the coloured area shows the 75% percentile, the middle bar depicts the median, and the lower bar shows the 25% percentile. Values that are above the 75% percentile and below the 25% percentile are shown outside the coloured area. Each individual data point represents plasma p-tau217 concentrations measured from a single participant within the respective age group.

In cohort-1, levels of plasma p-tau217 in newborns were significantly higher in the healthy newborns group (*n* = 55, 10.19 ± 3.92 pg/mL) compared with teenagers (*n* = 12, 1.79 ± 0.43 pg/mL), young adults (*n* = 28, 1.33 ± 0.69 pg/mL) and older adults (*n* = 28, 1.78 ± 1.31 pg/mL). No significant differences were observed in levels of plasma p-tau217 between teenagers, young and old adults (Fig. 1, *P* > 0.99). Moreover, the results observed in newborns in cohort-1 were replicated in cohort-2 (*n* = 106). No significant differences were observed between levels of plasma p-tau217 in newborns in cohort-1 and cohort-2 (10.19 ± 3.92 pg/mL versus 9.14 ± 4.87 pg/mL, *P* > 0.99, Fig. 1).

Cohort 1 included a set of participants with biomarker data for plasma NfL, total tau, Aβ42 and Aβ40, but without plasma p-tau217. Although direct comparisons with p-tau217 were not possible, we observed a similar pattern between plasma total tau (T-tau) and p-tau217, with both showing significantly higher levels in newborns compared to other groups (*P* < 0.0001; Fig. 1 and Supplementary Fig. 1). Conversely, plasma Aβ42 and Aβ40 levels were significantly lower in the newborn group compared to the other groups (*P* < 0.0001; Supplementary Fig. 1). Plasma NfL levels were significantly higher in newborns than in teenagers (*P* = 0.0002), yet lower than in older adults (*P* = 0.0027).

### Associations of cord blood p-tau217 with perinatal factors

Cohort-2 included extensive clinical data, enabling the exploration of associations between p-tau217 and perinatal factors. These findings build upon a previous publication that examined the relationship between Alzheimer’s disease biomarkers and neonatal hypoxia, where we reported that cord blood BD-tau is associated with indicators of foetal hypoxia, while p-tau217 levels may reflect a more general susceptibility to neurodevelopmental issues.^29^ In the present study, we contextualize these associations within biomarker changes observed in older individuals and Alzheimer’s disease.

In cohort-2, levels of p-tau217 in umbilical cord blood were negatively associated with gestational age (Spearman’s rho = −0.25, *P* = 0.0096), birth weight (Spearman’s rho = −0.23, *P* = 0.019) and head circumference (Spearman’s rho = −0.24, *P* = 0.016). However, only gestational age remained significant after multivariable analysis.^29^ Notably, data on cord BD-tau and NfL revealed no significant associations with perinatal factors.^29^ This distinction emphasizes the differences in dynamics between phosphorylated and non-phosphorylated forms of tau despite showing similar increases in newborns.

Additionally, while the method of delivery significantly influenced BD-tau and NfL levels—caesarean section being associated with the highest increases in these markers—no such variation was observed for p-tau217, which remained consistent across delivery methods.^29^

### Plasma p-tau217 levels in healthy controls, Alzheimer’s disease and newborns

To investigate how plasma p-tau217 concentrations compared between newborns and Alzheimer’s disease, we compared p-tau217 levels in newborns from cohort-1 (*n* = 55) and cohort-2 (*n* = 106) with controls (*n* = 56) and patients with Alzheimer’s disease (*n* = 163) from the Dementia Disease Initiation cohort (cohort-3).

**Figure 2:**
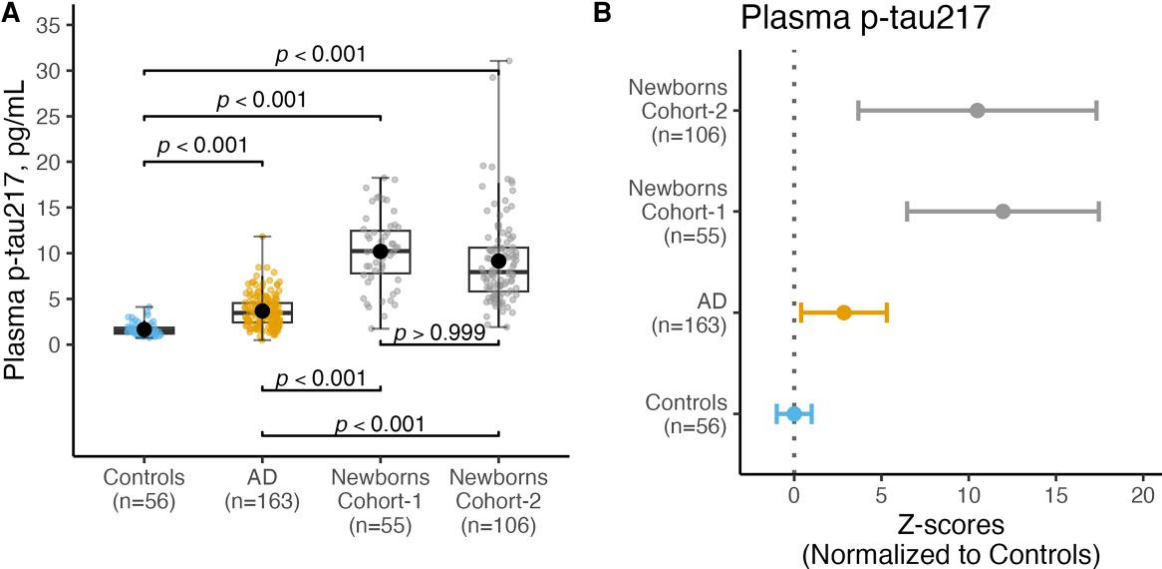
**Levels of plasma p-tau217 in healthy controls, Alzheimer’s disease and newborns. A** shows a comparison of plasma p-tau217 concentrations (pg/mL) among four groups: controls (*n* = 56), Alzheimer’s disease cohort (*n* = 163), newborns cohort-1 (*n* = 55) and newborns cohort-2 (*n* = 106). Group differences were examined using the Mann–Whitney test (two categories) or the Kruskal–Wallis test with Dunn’s multiple comparisons (three groups). In each box plot, the horizontal bar on top of the coloured area shows the 75% percentile, the middle bar depicts the median, and the lower bar shows the 25% percentile. Values that are above the 75% percentile and below the 25% percentile are shown outside the coloured area. Each individual data point represents the plasma p-tau217 concentration measured from a single participant within the respective group. (**B**) *Z*-score-transformed plasma p-tau217 concentrations are shown for the control group (cohort-3), the Alzheimer’s disease group (cohort-3) and newborns (cohort-1 and cohort-2). Group differences in this panel were also assessed using the Kruskal–Wallis test with Dunn’s multiple comparisons test.

The control group exhibited the lowest levels of plasma p-tau217 with concentration being similar to the ones in the older adults group in cohort-1 (1.66 ± 0.71 pg/mL versus 1.78 ± 1.31 pg/mL). In comparison, plasma p-tau217 levels in the Alzheimer’s disease group showed a significant increase (3.68 ± 1.75 pg/mL) and a high accuracy at differentiating Alzheimer’s disease from controls (Area under the curve: 0.88, Supplementary Fig. 2). However, levels of p-tau217 in newborns from cohort-1 (10.19 ± 3.92 pg/mL) and cohort-2 (9.14 ± 4.87 pg/mL) were significantly higher than levels in Alzheimer’s disease (3.68 ± 1.75 pg/mL). See Fig. 2A.

When using the control group from cohort-3 as reference, we observed the following fold changes, 2.8 for Alzheimer’s disease participants, 12 for newborns in cohort-1 and 10.54 for newborns in Cohort 2. These results are presented as *Z*-scores in Fig. 2B.

The lack of significant difference in p-tau217 levels between newborns in cohort-1 and cohort-2 (Fig. 2, *P* > 0.99), and between the older adults’ group in cohort-1 and the control group in cohort-3 (*P* > 0.99), indicates consistent plasma p-tau217 levels across the different cohorts.

### Longitudinal changes of serum p-tau217 in premature newborns

**Figure 3:**
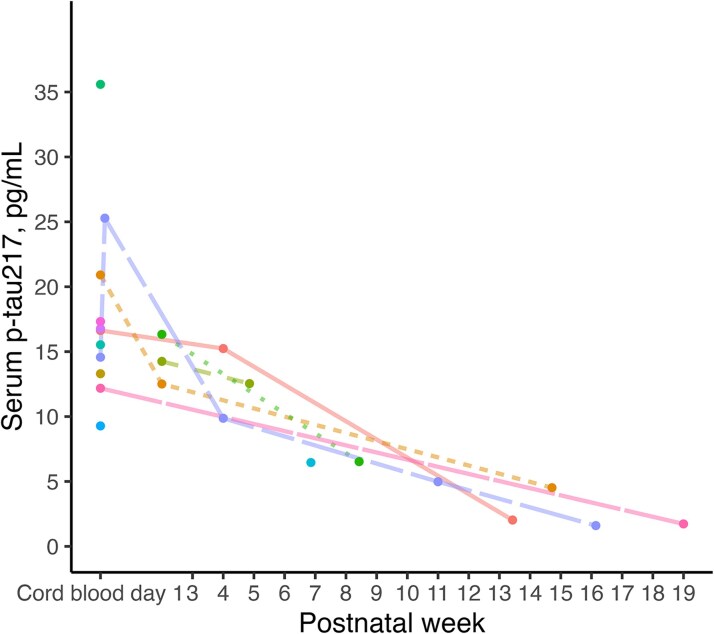
**Longitudinal trajectories of serum p-tau217 in preterm infants < 28 weeks gestational age at birth.** Spaghetti plot showing the longitudinal changes in serum p-tau217 levels in individual preterm infants over time (*n* = 14), measured in postnatal weeks. Each curve represents a single patient, with dots marking serum p-tau217 levels at specific time points.

In cohort-4, we evaluated longitudinal changes in serum p-tau217 levels in 14 extremely premature preterm newborns (<28 weeks gestational age) over time, measured in postnatal weeks. In Fig. 3, each curve represents a single patient, with dots marking p-tau217 levels at specific time points. Despite this overarching pattern, there is notable inter-patient variability in both the initial levels of p-tau217 and the rate of decline. Some newborns exhibit markedly high levels shortly after birth, with steep reductions, while others demonstrate lower starting levels and a more gradual decrease. By the later stages of postnatal development (Days 100–140), p-tau217 levels in all patients approach a stable, low range, suggesting the establishment of a baseline level as the brain matures.

## Discussion

Tau hyperphosphorylation not always leads to aggregation but rather seems to play a dual role in the brain.^33^ Phosphorylation sites like serine-262 have been widely studied in the context of brain development and more recently recognized as a critical epitope involved in tau pathology and tangle formation in Alzheimer’s disease,^9,11^ suggesting shared mechanisms in brain physiology and pathology. Here, we demonstrate that increased levels of phosphorylated tau can also be detected in the peripheral blood of newborns and that plasma p-tau217 levels are significantly higher in newborns compared to healthy individuals across different age groups. Importantly, longitudinal data show a decline in blood-based p-tau levels during the first months of life, eventually reaching levels similar to those in young adults. Moreover, in two independent cohorts, levels of p-tau217 in newborns were significantly higher than in Alzheimer’s disease. Our findings show that the increased levels of blood-based p-tau217 in newborns can be indicative of its physiological role in normal neurodevelopment.^2,4,34^ This is further supported by the associations between umbilical cord blood p-tau217 levels and perinatal variables such as gestational age that underscore the relevance of tau phosphorylation in foetal brain development.^4^ This feat was unique to phosphorylated tau in our data set since we observed no associations between perinatal variables and non-phosphorylated tau markers, such as BD-tau, or NfL.^29^

In line with these results, longitudinal measurements of serum p-tau217 in premature newborns revealed that this marker remains elevated for several weeks after birth before declining towards a ‘baseline level’ found in young adults. This persistence aligns with the dynamic regulation of tau phosphorylation during the early stages of postnatal brain development, potentially driven by ongoing neuroplasticity and the maturation of enzymatic pathways involved in tau regulation.^10,11^ The variability in decline rates among individuals further points to the influence of inter-patient differences in developmental trajectories. These differences in trajectories might reflect the dynamic regulation of tau phosphorylation during early development, likely driven by increasing maturation of kinases, phosphatases and the increased expression of adult over foetal isoforms.^2^ The variability in the decline rates and initial levels may be influenced by differences in perinatal factors, as observed in cohort-2.

The markedly higher p-tau217 levels with no aggregation or tangle formation in newborns compared to Alzheimer’s disease suggest distinct physiological processes governing tau phosphorylation during development versus neurodegenerative disease.^2^ It is still unknown if different p-tau markers show different increases and trajectories in newborns like they do in Alzheimer’s disease. Our findings suggest a unique regulatory role for p-tau in neurodevelopment that warrants further investigation.^2,34^ While our results show clear increases in blood-based p-tau217 levels in both healthy and premature newborns, the specific contributions of foetal tau versus adult tau isoforms to the detected blood levels remain unclear, as tau immunoassays target regions shared by both foetal and adult isoforms. Understanding the mechanisms of maintaining high p-tau levels without triggering aggregation or pathology during foetal development could offer valuable insights for Alzheimer’s disease and other tauopathies.^35^ The natural protective mechanisms in newborns may be mimicked as therapeutic approaches aiming at modulating tau phosphorylation, improving tau clearance and preventing pathological tau aggregation in neurodegenerative diseases. Future studies exploring the individual contributions of the different tau isoforms to the elevated phosphorylated tau levels in blood and the protective mechanisms that prevent aggregation could lead to novel interventions that replicate these developmental conditions in Alzheimer’s disease.

## Strengths and limitations

This is the first report comparing levels of plasma p-tau217 in newborns, controls and Alzheimer’s disease using currently available blood-based immunoassays. Strengths of this study include the multicentric evaluation of blood-based p-tau217 and the inclusion of different age groups and two large cohorts with newborn samples. However, there are important limitations that should be acknowledged, including the lack of longitudinal plasma p-tau217 data and the lack of comparisons with other biomarkers across all the cohorts included in this study. Additionally, future studies should corroborate our results by using different immunoassays and platforms apart from Simoa. Our study provides an impetus to explore phosphorylated tau biomarkers in cord blood.

## Supplementary Material

fcaf221_Supplementary_Data

## Data Availability

No new software and/or algorithms, in-house scripts or programme were generated to support this study. Requests for the data sets used in the present study will be promptly reviewed by the corresponding authors and the University of Gothenburg to verify whether the request is subject to any intellectual property or confidentiality obligations. Anonymized data can be shared by request from any qualified investigator for the sole purpose of replicating procedures and results presented in the article, if data transfer is in agreement with EU legislation. All requests for code used for data analyses and data visualization will be promptly reviewed by the corresponding author and the University of Gothenburg.
