# Supplementary material for: The potential dual role of tau phosphorylation: plasma phosphorylated-tau217 in newborns and Alzheimer’s disease
Source: Brain Commun. 2025 Jun 7;7(3):fcaf221. doi: 10.1093/braincomms/fcaf221 (PMC12198956; doi:10.1093/braincomms/fcaf221)
Supplement: fcaf221_Supplementary_Data [file fcaf221_supplementary_data.docx]

**The potential dual role of tau phosphorylation: plasma phosphorylated-tau217 in newborns and Alzheimer's disease**

**Authors:** Fernando Gonzalez-Ortiz^1,2,3#^; Jakub Vávra^1^; Emma Payne^4^; Bjørn-Eivind Kirsebom^5,6,7^; Ulrika Sjöbom^8,9^; Cristiano Santos^2^; Jordi Júlvez^10^; Kaitlin Kramer^11,12^; David Zalcberg^11,12^; Laia Montoliu-Gaya^1^; Michael Turton^13^; Peter Harrison^13^; Ann Hellström^9^; Henrik Zetterberg^1,2,14,15,16,17^; Tormod Fladby^7,18^; Marc Suárez-Calvet^19,20,21^; Robert D. Sanders^11,12,22,23^; Kaj Blennow^1,2,24,25^

**Author affiliations:**

1. Department of Psychiatry and Neurochemistry, Institute of Neuroscience and Physiology, the Sahlgrenska Academy at the University of Gothenburg, Gothenburg, Sweden
2. Clinical Neurochemistry Laboratory, Sahlgrenska University Hospital, Mölndal, Sweden
3. Neurocode USA Inc, 3560 Meridian St, Bellingham, WA 98225, United States
4. St George Hospital, South Eastern Sydney Local Health District, Sydney, New South Wales, Australia
5. Department of Neurology, University Hospital of North Norway, Tromsø, Norway
6. Department of Psychology, Faculty of Health Sciences, The Arctic University of Norway, Tromsø, Norway
7. Department of Neurology, Akershus University Hospital, Lørenskog, Norway
8. Learning and Leadership for Health Care Professionals at the Institute of Health and Care Science at Sahlgrenska Academy at University of Gothenburg, Gothenburg, Sweden
9. Department of Clinical Neuroscience at the Institution of Neuroscience and Physiology at Sahlgrenska Academy at University of Gothenburg, Gothenburg, Sweden
10. Clinical and Epidemiological Neuroscience Group (NeuroÈpia), Institut d'Investigació Sanitària Pere Virgili (IISPV), 43204, Reus, Spain
11. Central Clinical School, Faculty of Medicine and Health, The University of Sydney, Sydney, New South Wales, Australia
12. Department of Anaesthetics, Royal Prince Alfred Hospital, Sydney Local Health District, Sydney, New South Wales, Australia
13. Bioventix Plc, 7 Romans Business Park, East Street, Farnham, Surrey GU9 7SX, UK
14. Department of Neurodegenerative Disease, UCL Institute of Neurology, Queen Square, London, UK
15. UK Dementia Research Institute at UCL, London, UK
16. Hong Kong Center for Neurodegenerative Diseases, Clear Water Bay, Hong Kong, China
17. Wisconsin Alzheimer’s Disease Research Center, University of Wisconsin School of Medicine and Public Health, University of Wisconsin-Madison, Madison, WI, USA
18. University of Oslo, Institute for Clinical Medicine, Campus Ahus, Oslo, Norway.
19. Barcelonaβeta Brain Research Center (BBRC), Pasqual Maragall Foundation, Barcelona, Spain.
20. Hospital del Mar Research Institute, Barcelona, Spain.
21. Servei de Neurologia, Hospital del Mar, Barcelona, Spain.
22. NHMRC Clinical Trials Centre, The University of Sydney, Sydney, New South Wales, Australia
23. Institute of Academic Surgery, Royal Prince Alfred Hospital, Sydney Local Health District, Sydney, New South Wales, Australia
24. Paris Brain Institute, ICM, Pitié-Salpêtrière Hospital, Sorbonne University, Paris, France
25. Neurodegenerative Disorder Research Center, Division of Life Sciences and Medicine, and Department of Neurology, Institute on Aging and Brain Disorders, University of Science and Technology of China and First Affiliated Hospital of USTC, Hefei, P.R. China

#**Correspondence:** Fernando Gonzalez-Ortiz, Clinical Neurochemistry Lab House V3, floor 2 Mölndal Hospital Street Address: Biskopsbogatan 27 SE-43180 Mölndal, Sweden.

Email: [fernando.gonzalez.ortiz@gu.se](mailto:fernando.gonzalez.ortiz@gu.se)

**Running title:** Plasma p-tau217 is higher in newborns than in AD

**Key words:** phosphorylated tau; newborns; plasma biomarkers; Alzheimer's disease

| **Supplementary Table 1** Demographics for cohort- 4 | |
| --- | --- |
| **P*reterm infants***  ***(14)*** | |
| **Gestational Age (weeks)** Mean (SD) | 26.2  (1.3) weeks |
| **Female** n (%) | 3 (21.4) |
| ***Serum p-tau217 pg/mL at birth (cord blood)*** Mean (SD) | 16.7  (7.7) |
| Abbreviations SD, standard deviation; n, number of cases; %, percentage | |

| **Supplementary Table 2.** Demographics and biomarker concentrations for other plasma markers in cohort-1 | | | | |
| --- | --- | --- | --- | --- |
| ***Cohort-1 (ALFA age)*** | | | |  |
|  | **Newborns** | **Teenagers** | **Young adults** | **Older adults** |
| **^a^Gest. Age / ^b^Age Years** Mean (SD) [range] | ^a^39.5 (0.8) [38 – 41] | NA | ^b^23.0 (2.0) [19 – 26] | ^b^74.8 (3.3) [71 – 85] |
| **Female** n (%) | 8 (34.8) | NA | 26 (86.7) | 17 (60.7) |
| ***Plasma Aβ1-40, pg/ml*** | 17.8 (17.2) [n= 28] | 214.0 (48.1) [n= 30] | 140,6 (37.7) [n= 30] | 180.4 (42.2) [n= 30] |
| ***Plasma Aβ1-42, pg/ml*** | 2.1 (3.5) [n= 29] | 13.9 (3.5) [n= 31] | 9.3 (2.4) [n= 30] | 10.4 (2.4) [n= 28] |
| ***Plasma Aβ42/40 ratio*** | 0.13 (0.11) [n= 27] | 0.07 (0.01) [n= 30] | 0.7 (0.01) [n= 30] | 0.06 (0.01) [n= 28] |
| ***Plasma NfL, pg/ml*** | 10.5 (4.2) [n= 25] | 5.4 (2.4) [n= 31] | 8.1 (6.1) [n= 30] | 21.9 (8.3) [n= 30] |
| ***Plasma t-tau, pg/ml*** | 28.4 (11.07) [n= 30] | 4.4 (1.7) [n= 31] | 2.8 (1.1) [n= 30] | 3.0 (1.6) [n= 30] |
| Abbreviations: n, number of cases; %, percentage; a, Gestational age; b, Years of age | | | | |


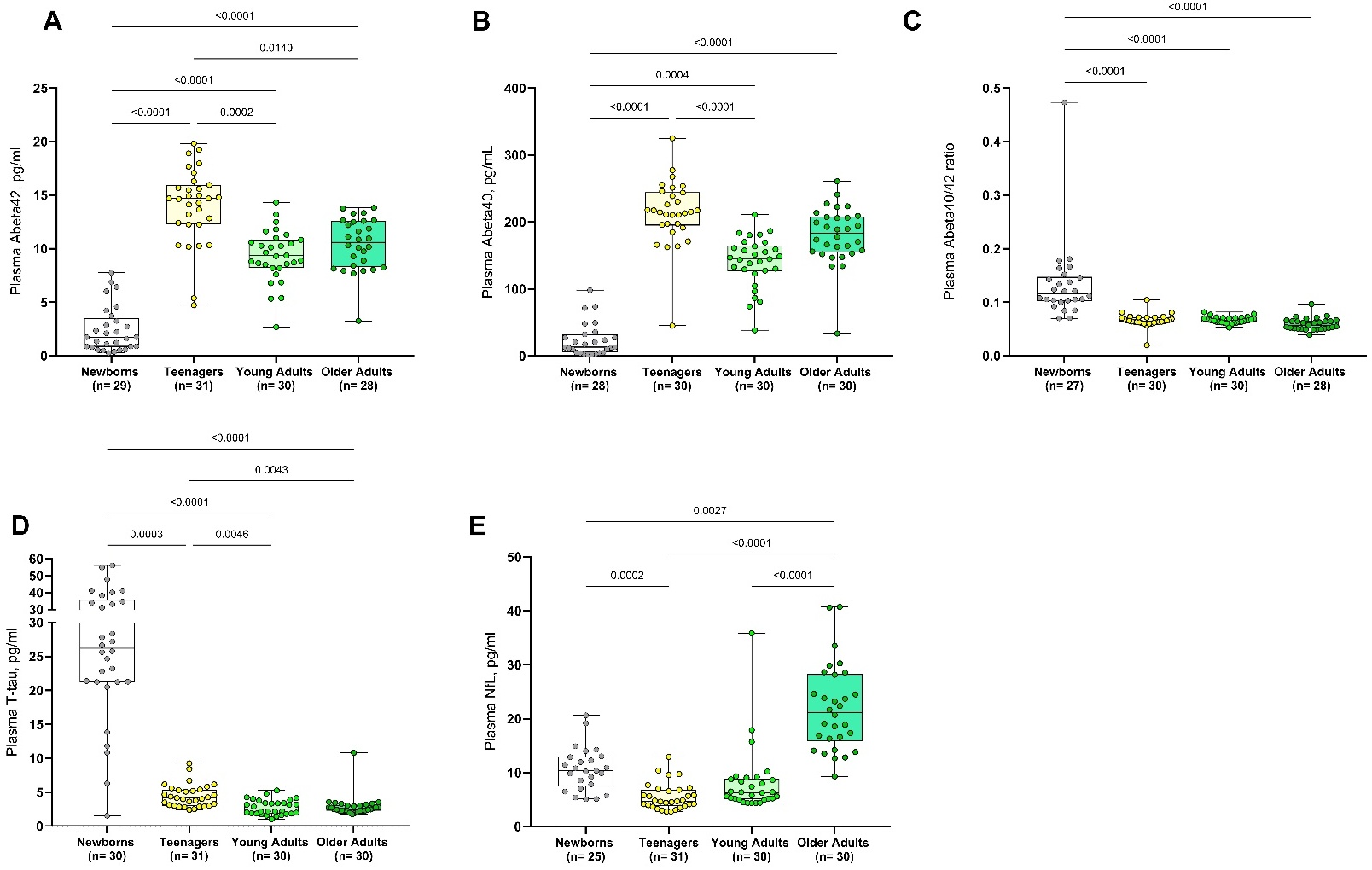


**Supplementary Figure 1**. Plasma NfL, T-tau and amyloid40 and amyloid42 in cohort 1. P-values indicate the results of Mann–Whitney test (for two groups) or Kruskal–Wallis test adjusted for multiple comparisons (three or more groups). In each box plot, the horizontal bar on top of the colored area shows the 75% percentile, the middle bar depicts the median and the lower bar shows the 25% percentile. Values that are above the 75% percentile and below the 25% percentile are shown outside the colored areas. Each individual data point represents the corresponding biomarker concentration measured from a single participant within the respective age group.


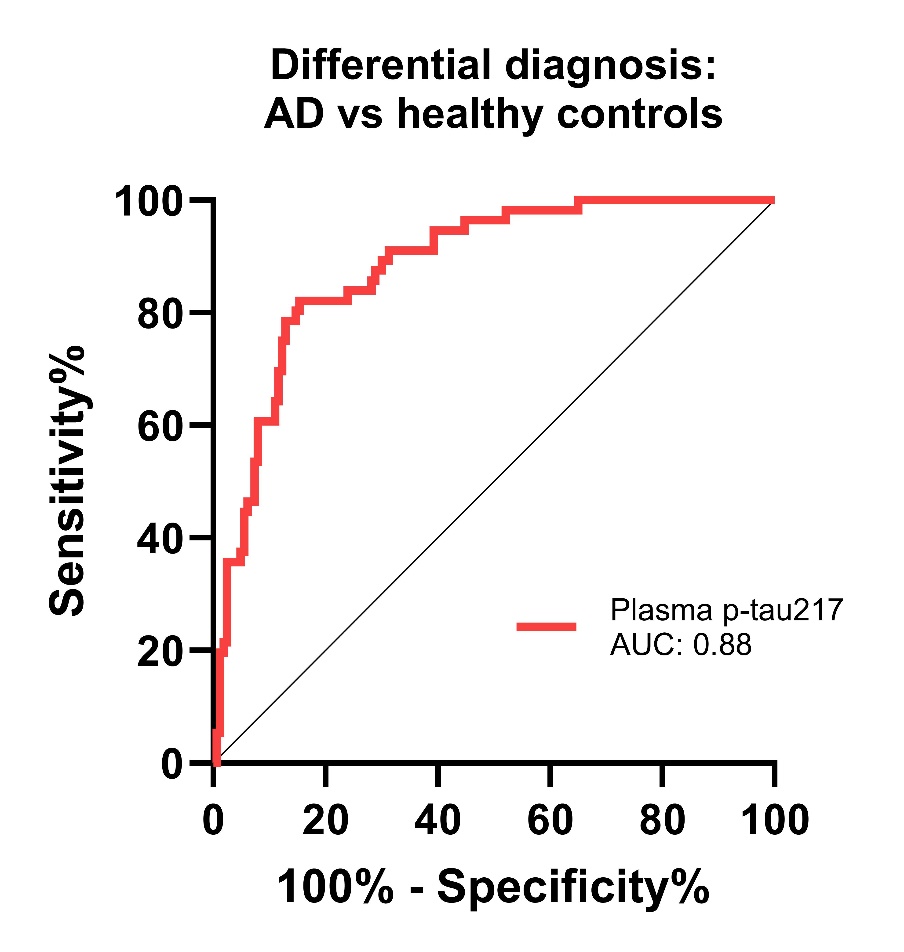


**Supplementary Figure 2**. Diagnostic accuracy of plasma p-tau217 in cohort-3. Receiver operating curve (ROC) and area under the curve (AUC) values indicating between-group discriminatory accuracies of plasma p-tau217 in controls (n=56) and patients with AD (n=163) in the Dementia Disease Initiation cohort (cohort-3). The diagonal line on the ROC plot shows 50% accuracy meaning no difference from chance events. The analysis included
